# Mendelian Randomization — the Key to Understanding Aspects of Parkinson's Disease Causation?

**DOI:** 10.1002/mds.26492

**Published:** 2015-12-23

**Authors:** Alastair J. Noyce, Mike A. Nalls

**Affiliations:** ^1^ Reta Lila Weston Institute for Neurological Studies, UCL Institute of NeurologyLondonUK; ^2^ Laboratory of Neurogenetics, National Institute on Aging, National Institutes of HealthBethesdaMarylandUSA

**Keywords:** Parkinson's disease, Mendelian randomization, observational study, causation

## Abstract

Parkinson's disease has multiple determinants and is associated with a wide range of exposures that appear to modify risk in traditional observational studies, including numerous lifestyle and environmental factors. Across other fields of medicine, Mendelian randomization has emerged as a powerful method to examine whether associations between exposures and disease outcomes are causal. Here we discuss the concept of Mendelian randomization, its potential relevance to Parkinson's disease, and suggest avenues through which the method could be employed to further understanding of the causal basis of Parkinson's disease. © 2015 The Authors. Movement Disorders published by Wiley Periodicals, Inc. on behalf of International Parkinson and Movement Disorder Society.

Two decades ago, Parkinson's disease (PD) was thought to be an entirely sporadic disorder with little, if any, genetic component. Despite identification of monogenic forms of disease (such as mutations in *SNCA, LRRK2, PINK1, PARK2*, and *DJ‐1*)^1^, ^2^, ^3^ and genetic risk factors for PD (such as mutations in *GBA*),^4^, ^5^, ^6^ this is a prevailing view held by some clinicians and much of the patient community. Up to 10% of patients report a family history of PD,^7^ but family history is different from heritable genetic risk. The genetic architecture of PD is complex, and genetic variability likely accounts for a large proportion of the heterogeneity of PD, even if much of this currently remains unexplained. Conservative heritability estimates attributable to common genetic risk in PD are approximately 30%, even in sample series with only limited family history.^8^

The monogenic forms of PD, identified through candidate gene studies, are individually sufficient to cause PD, but account for a small proportion of PD caseload overall (∼5%).^2^ Separately, genome‐wide association studies (GWAS) have identified common, low‐effect variants that account for additional heritability, each potentially acting in a small but additive fashion, and 28 independent susceptibility loci for PD have been reported.^9^ Meta‐analyses of GWAS have supported some of the findings of candidate gene work and provided estimates of effect size for a range of variants, with some of the larger ones being *LRRK2* (odds ratio [OR] for G2019S mutation, 9.62; 95% confidence interval (CI), 6.43‐14.37)^10^ and *GBA* (N370S mutation OR, 3.51; 95% CI, 2.55‐4.83).^11^

Environmental risk factors for PD have tended to be associated with small effect sizes in observational studies (OR, 1.2‐1.8).^12^ In some cases even these may have been artificially inflated by bias and/or confounding (see Fig. 1). Confounding by prevalent disease (reverse causality) is a particular problem for many factors associated with reduced risk of PD, including cigarette, coffee, and alcohol consumption, given that patients may exhibit specific personalities in the years before diagnosis.^13^ Thus, apparent temporal associations with exposures may have been identified, when in fact they are indicative of prodromal or prediagnostic disease, rather than being causally related. This issue may have applied to cohort studies that excluded incident cases within the first few years of follow‐up because the prodromes of PD are likely to be very long.^14^ For factors that are associated with elevated risk of PD, such as head injury caused by falling because of undiagnosed prevalent disease (as opposed to historical sports or combat‐related injuries), the same might apply. The biases of retrospective case‐control studies are well recognized, including but not limited to recall bias, selection bias, and observer bias.

**Figure 1:**
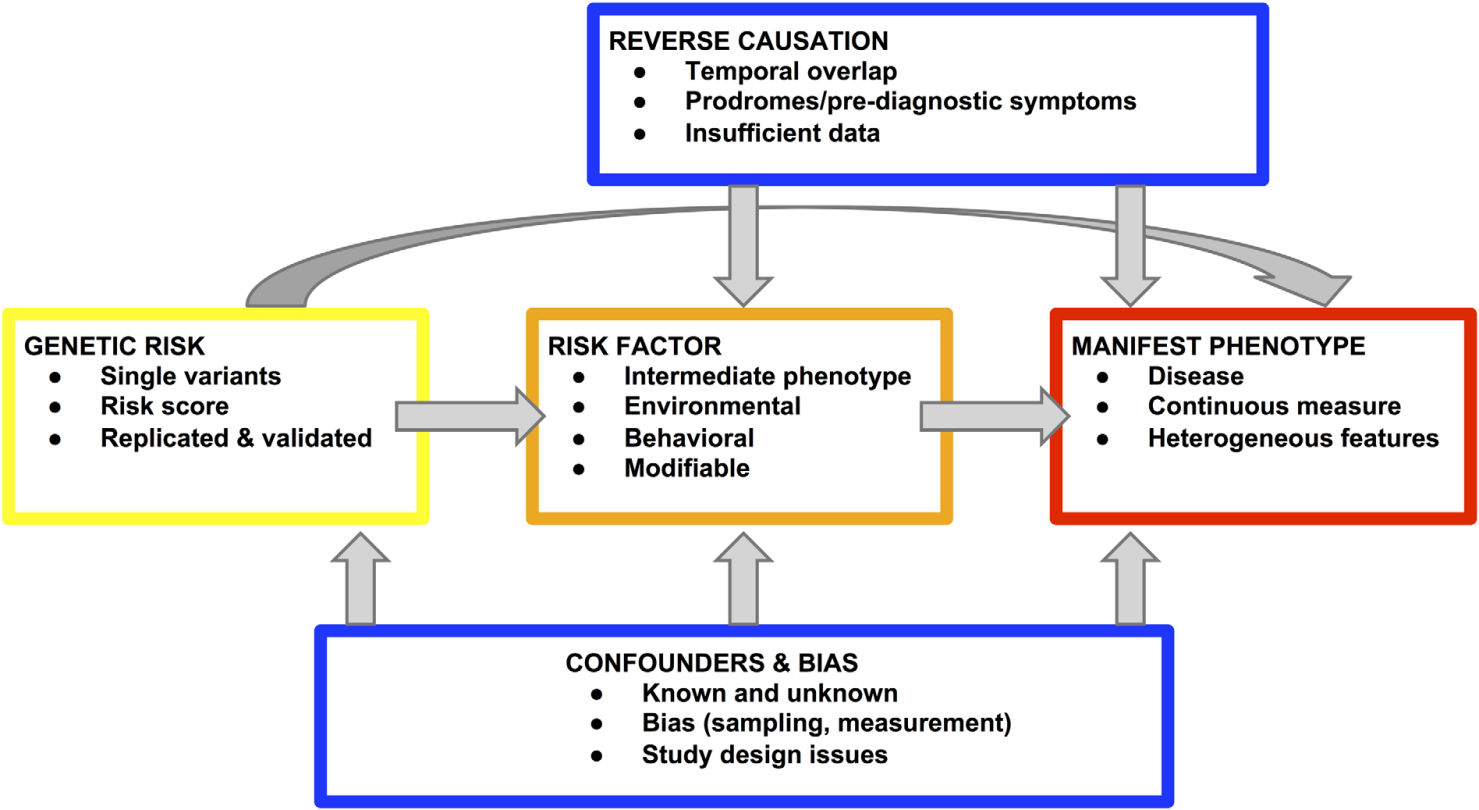
Genetic (yellow) and nongenetic (orange) risk factors, their influence on phenotype (red), and where confounding, bias and reverse causality can arise (blue). [Color figure can be viewed in the online issue, which is available at wileyonlinelibrary.com.]

Thus, when inferring causality, well‐conducted randomized controlled trials (RCTs) sit at the head of a hierarchy, superior to observational study designs, which follow beneath. If sample sizes are large, the process of allocating an exposure (intervention or drug) to patients on a sufficient scale, randomly and evenly distributes known and unknown confounding factors, meaning that any effect observed can be attributed to causal influence. Furthermore, the effect is free from reverse causality and sheltered from bias (as long as the selection process and blinding have been optimized).

Opportunities to use RCTs to study causal relationships between environmental exposures and PD are limited by the ethical and logistical challenges they pose (see Table 1). For example, it is not acceptable to expose subjects to smoking and pesticides for the purpose of causal inquiry, and it is difficult to ensure that randomization to nonsteroidal anti‐inflammatory drugs (NSAIDs) versus no NSAIDs would be upheld when they are available over the counter.

**Table 1:** Recognized risk and protective factors for PD,^12^ the nature of association, and limitations when considering randomized controlled trials

Factor	Evidence	Association with risk of PD	Problem(s) with assessing causal effect using RCT design
Smoking	+++	Protective	Negative effects on wider health
Alcohol	+++	Protective	Negative effects on wider health
NSAIDs	++	Protective	Difficult to prevent subjects in placebo arm from using over‐the‐counter forms
Statins	++	Protective	None — investigational medicinal product in an RCT (*personal communication with principal investigator*)
CCBs	+++	Protective	None — investigational medicinal product in an RCT (clinicaltrials.gov reference NCT02168842)
Coffee	+++	Protective	Difficult to prevent subjects in placebo arm from consuming
Pesticides	+++	Increase risk	Heterogeneous, measurement
Head injury	+++	Increase risk	Reverse causality (ie, head injury from falls), long lag time (ie, head injury from sports)
Obesity	+/‐	Increase risk	Measurement, masking by prevalent disease, negative effects on wider health
Heavy metals	+/‐	Increase risk	Heterogeneous, measurement Iron — investigational medicinal product in an RCT (clinicaltrials.gov reference NCT01539837)
Low serum urate	++	Increase risk	Under investigation (clinicaltrials.gov reference NCT00833690)

^+++^Meta‐analysis of observational studies; ^++^multiple observational studies suggesting directionality of effect; ^+/‐^conflicting data from single studies. RCT, randomized, controlled trial; PD, Parkinson's disease; NSAID, nonsteroidal anti‐inflammatory drugs; CCB, calcium channel blockers.

In other fields of medicine, Mendelian randomization (MR) has emerged as a technique for gaining greater understanding of the role that environmental exposures play in disease causation.^15^, ^16^ Here genetic variants are used as so‐called ‘instrumental variables’ that reflect a particular environmental exposure by virtue of attesting to, in a dose‐dependent manner, differential exposure status (for example, a genetic variant associated with obesity may convey an increase in body mass index per allele inherited). Alleles are allocated at conception and operate with long‐lasting effects, which negate the risk of reverse causality. However, gene expression may vary through life, meaning that detailed biological understanding can hugely enhance inferences made from MR. Random assortment during meiosis can be seen as equivalent to the randomization process in a well‐conducted RCT; moreover, the random allocation of all other genetic material is akin to the random distribution of known and unknown confounding variables alongside exposure status. The causal inference that can be drawn from a well‐conducted MR study sits below that of well‐conducted RCTs and above observational study designs, as long as limitations are recognized and understood (see Table 2 for advantages and disadvantages).

**Table 2:** Advantages and limitations of MR in inferring causality (adapted from Lawlor et al^16^)

Advantages	Disadvantages and limitations
Random allocation of genetic variant of interest (and therefore exposure) — avoids selection bias and is synonymous with methods used for traditional RCTs.	Pleiotropy — horizontal pleiotropy describes the situation in which a genetic variant affects the outcome via a different pathway from the one that includes the exposure under investigation and may result in bias. There are ways to avoid/negate pleiotropy, such as the selection of multiple instrumental variables, or correct the bias that it creates, such as the use of MR‐Egger.^26^
Random allocation of confounders — also avoids selection bias and evenly distributes confounding factors between exposed and unexposed (as per traditional RCTs).	Population stratification — different populations may have different rates of disease (outcome of interest) and/or different distributions of genetic variants (exposure of interest), resulting in biased effect estimates.
Blinding — patient does not know his/her genetic variant (exposure) status and therefore behavior is unlikely to be affected (as per traditional RCTs).	Linkage disequilibrium (LD) — gene loci are presumed to be independent of one another, but LD describes the situation in which they are not; genetic variants may be coinherited, and bias can result. This can create a situation in which horizontal pleiotropy or confounding occurs. However, in some instances, LD is an advantage and allows an unmeasured variant, which influences the exposure of interest, to be estimated through use of a measured gene variant as a proxy.
Life‐long exposure — effects may be exerted throughout life as long as expression not differential (significant advantage over traditional RCTs).	Canalization — describes developmental compensation to neutralize the effect of a particular genotype on a disease outcome, but not necessarily the association with the exposure. This may significantly weaken the instrumental variable and bias estimates.
Ethically sound — allows study of the potential effect of exposures that are not appropriate for traditional RCTs ie, smoking.	Statistical power — the magnitude of effect that allelic increases have on an environmental exposure is often very small (and may be tiny if the instrument is weak). This means that sample sizes must be very large.
Cost effective — compared with RCTs or prospective cohort studies. MR studies can often be conducted in situations in which much of the data (genetic and clinical) has already been collected.	Adequate biological understanding — detailed information is required about the effects of the variant of interest, conditions under which it is expressed, or conditions that alter expression to make inferences about causality.

A number of criteria^17^ must be satisfied to identify genetic variants as suitable instrumental variables for environmental exposures (depicted in Fig. 2):

1. The genetic variant must be robustly associated with the exposure.
2. The genetic variant must be independent of known confounders.
3. For a known exposure and known confounders, the genetic variant must be independent of the outcome (known as the exclusion restriction criteria). In other words, there must no alternative route, other than via the exposure, that the instrumental variable is associated with the outcome.

**Figure 2:**
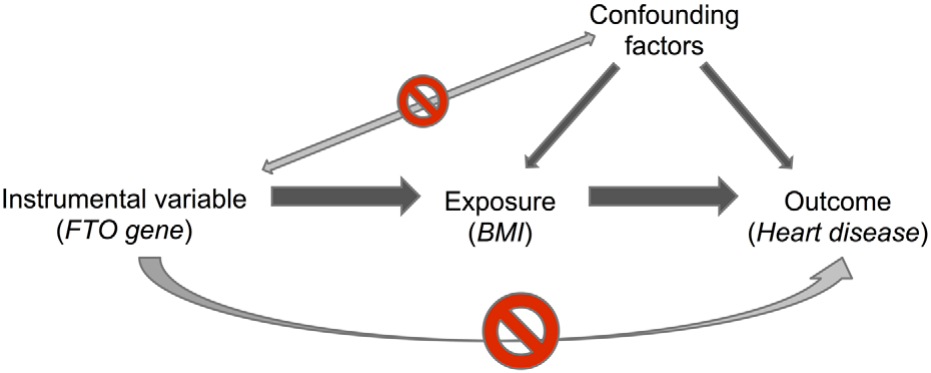
Directed acyclic graph (DAG) demonstrating the concept and assumptions of Mendelian randomization (with an example in parentheses), including the relationship of the instrumental variable with the exposure (assumption 1) and independence from confounding factors (assumption 2) and the outcome (assumption 3). Note that the only way that the instrumental variable influences the outcome is via the exposure (behavior or phenotype). In the example given, the *FTO* gene variant (instrumental variable) influences BMI (exposure/intermediate phenotype), which is causally associated with heart disease (outcome). [Color figure can be viewed in the online issue, which is available at wileyonlinelibrary.com.]

Searching the Medline database provides sparse examples of the use of MR in PD (3 articles found using the search terms “Mendelian randomization” and “Parkinson's disease”) and, indeed, all neurological disease to date, apart from those undertaken in the study of stroke.^18^, ^19^ However, a group recently reported observations from an MR study in subjects with PD. The *a priori* hypothesis was that, consistent with findings from observational studies, low serum urate was associated with increased risk of PD. The authors hypothesized that polymorphisms that predict serum urate levels would also predict rate of clinical progression in subjects with early PD from the PRECEPT and DATATOP studies. They selected single‐nucleotide polymorphisms (SNPs) within the *SLC2A9* gene, which explains most of the genetically specified variance in serum urate, and a dose‐dependent reduction in serum urate for increasing allele counts was observed.^20^ There was weak evidence for an increased rate of disease progression, defined as disability requiring dopaminergic treatment (increasing *SLC2A9* score HR, 1.16; 95% CI, 1.00‐1.35; *P* = 0.06). Directionality of effect was consistent between measured decreases in serum urate or genetically conferred decreases. Stronger effects may have been masked by the choice of outcome and the modest sample size of ∼800 subjects, but these results increase confidence that targeting elevations in urate may be a plausible approach in neuroprotective studies in PD. Other studies have previously considered genetic influence on serum urate levels and PD risk, as well as interactions between variants and environmental factors, but not specifically adopting MR approaches.^21^, ^22^ These may have violated some of the inherent assumptions of MR, have been underpowered, or require further replication.

Separately, a group reported observations relating to serum iron and PD, using data from PD GWAS.^23^ Three genetic variants were used as instrumental variables (*HFE* rs1800562, *HFE* rs1799945, and *TMPRSS6* rs855791) and were associated with serum iron levels (accounting for 1.7%, 0.9%, and 1.7% of iron total variance, respectively). Each variant conveyed an increase of iron level per allele of between 7.1 and 13.9 µg/dL. The polymorphism in *TMPRSS6* alone was associated with reduced risk of PD OR of 0.97 (95% CI, 0.94‐0.99; *P* = 0.034). Meta‐analysis to determine a pooled effect size across these 3 variants gave an OR of 0.88 (95% CI, 0.82‐0.95; *P* = 0.001) for the reducing the OR for PD per SD unit increase in iron, consistent with a protective effect.

In an exploratory study, another group used SNPs from the *CHRNA3‐CHRNA5‐CHRNB4* gene cluster known to be associated with smoking behavior to assess associations with PD.^24^ Although no effect on PD was found, the sample sizes were moderate, and the study may have been underpowered to detect an effect.

These examples introduce some of the potential limitations of MR, which include pleiotropy and statistical power. Horizontal pleiotropy describes the situation in which a genetic variant has multiple independent effects that do not lie on a single pathway, meaning that instrumental variable assumptions may be violated (see Table 2). Previously, use of multiple gene variants known to operate via the same intermediate phenotype (such as the example above from Pichler and colleagues^23^) and finding similar effect size estimates and absence of heterogeneity offered some evidence against pleiotropy.^25^

Post‐GWAS, increasingly, MR studies use multiple genetic variants to improve precision of causal effect estimates. This in turn can increase the risk of horizontal pleiotropy and bias. Adaptation of traditional meta‐analysis methods such as funnel plots to detect a difference in effect estimates because of weaker variants and MR‐Egger (based on Egger regression used to assess small‐study bias in meta‐analyses) can be used to detect and adjust for pleiotropy arising through multiple variant analysis.^26^

Away from pleiotropy, MR studies need to be sufficiently powered so that causal hypotheses may be adequately tested. At least 2 of the 3 MR studies in PD listed above may have been underpowered because of moderate sample sizes and may have failed to reject the null hypothesis, when in fact a real effect was present (type 2 error). Additional limitations include population stratification, canalization and adequate understanding of the underlying biology (all described in Table 2 along with potential implications).

Special reference should be made to the potential implications of linkage disequilibrium (LD). Gene loci are presumed to be independent of one another, but LD describes the situation in which they are not, and therefore genetic variants may be coinherited. Loss of independence is not exclusively a result of the proximity of loci, and coinheritance can occur despite loci being physically distant from one another. It is easy to see how this situation could negatively affect MR studies; however, this is not always the case, and many MR studies use LD to their advantage, using a measurable variant as proxy for an unobservable variant that influences the modifiable exposure. In other situations, LD can cause confounding and induce pleiotropy, which may in turn violate instrumental variable assumptions. The relevance of LD in MR studies has been discussed elsewhere and examples provided,^16^ but in summary, LD needs to be understood within the context of individual MR studies before deciding how it ought to be handled.

In many instances, there may not yet be sufficient variance explained by recognized gene variants to allow an MR approach to be undertaken for certain potentially modifiable exposures. However research in PD genomics moves forward with great momentum, and the onus to provide robust instrumental variables for modifiable behaviors such as smoking and alcohol use comes from even more powerful spheres of cardiovascular and cancer research. Within specific MR studies, the magnitude of association between the instrumental variable and the exposure ought to be established at the outset and confirmed to be consistent with estimates from independent studies. In addition, traditional confounders of the relationship between exposure and the outcome, should be described for each gene variant used in the study and shown to be no different between them rather like the baseline characteristics table used when reporting an RCT. In carrying out the MR study, it may be important to be able to compare the effect that the gene variant has on the outcome (via the exposure), with the effect that the directly measured exposure has on the outcome in the same cohort. Effects operating in the same direction give credence to the results from observational studies, whereas opposing effects are likely to warrant further investigation. The methodological approaches for undertaking MR studies are varied and numerous, and have been recently described.^27^

At present there exist a number of options to exploit MR and understand more about the variance of PD attributable to modifiable exposures (examples are listed in Table 3). For all the examples given, there exists extensive observational study literature and for caffeine, some limited RCT data.^28^ In addition, each of these factors has potential instrumental variables available, and MR could significantly advance understanding of their causal association with PD.

**Table 3:** Examples of variants that could be further explored as instrument variables to ascertain causal association of environmental factors and PD

Factor	Candidate variant examples	Anticipated effect
Smoking	rs1051730, rs4105144, rs6474412, rs8034191, rs17486278, rs569207, rs16969968, rs578776, rs6495308^24^, ^30^	Reduce/increase smoking quantity, increase/reduce risk of PD
Coffee	rs2472297‐T, rs6968865‐T^31^	Increase coffee consumption, reduce risk of PD
Alcohol	Alcohol dehydrogenase 1B gene (*ADH1B*)^19^	Reduce alcohol consumption, increase risk of PD
Obesity	*FTO* gene rs9939609 polymorphism^32^	Elevate BMI, increase risk of PD

Example variants identified from published literature and National Human Genome Research Institute (NHGRI) catalogue.

Traditional RCTs have their own limitations, including high cost and resource requirements, and in the context of PD, long follow‐up and in some cases impracticality. Hence, large‐scale prospective, observational studies and/or the mining of medical records and biosamples from massive health systems with electronic medical records could act as an in silico laboratory for MR studies and function much like an RCT with comparatively minimal bias. The key issue here is the quality and depth of phenotypic data available, the absence of which to‐date has meant limited attempts to make genotype‐phenotype assertions in large‐scale studies, with analyses restricted to comparing cases versus controls or genetic risk profiles for intermediate phenotypes and not the phenotypes themselves.^29^ We propose that in the context of improving phenotypic data and refining identification of functional genetic variants, focusing some resources to the field of MR could help to drive the understanding of PD causality significantly further forward.

## Summary

Other fields of medicine have seen a rapid expansion in the use of MR for etiological inquiry. We believe that MR has great potential to be used to further explore the causal basis for a variety of exposures and associations with PD that have been reported in observational studies. Many of these exposures do not naturally lend themselves to traditional RCTs because these would be considered unethical. However, confirmation of direction and magnitude of effects in MR studies with those from observational studies may prompt further consideration of these factors as targets for intervention and potentially disease modification.

## Author contributions

A.J.N. and M.A.N. were responsible for drafting and revising the manuscript.

## Financial disclosures of all authors (preceding 12 months)

A.J.N. has received grants from Élan/Prothena Pharmaceuticals and from GE Healthcare. M.A.N. reports no disclosures.
